# Evaluating Compressed SENSE (CS) MRI Metal Artifact Reduction Using Pig L-Spine Phantom and Transplant Patients: Focused on the CS-SEMAC (SPIR), mDixon(O-MAR) and STIR Techniques

**DOI:** 10.3390/tomography8050192

**Published:** 2022-09-15

**Authors:** Eun-Hoe Goo, Sung-Soo Kim

**Affiliations:** 1 Department of Radiological Science, Cheongju University, Cheongju 28503, Korea; 2 Department of Health Administration and Healthcare, Cheongju University, Cheongju 28503, Korea

**Keywords:** compressed SENSE, mDixon, SEMAC

## Abstract

This study evaluates the clinical usefulness of the images obtained after applying mDixon (O-MAR), CS-SEMAC (SPIR), and STIR techniques to Pig L-Spine Phantom and transplant patients according to the difference in the reduction in metal artifacts and provides the optimal MAR image technique. This study was conducted with Phantom and 30 transplant patients who had an implant on the L-Spine (22 men, 8 women, mean age: 64.2 ± 12.98). All data analyzed were evaluated, using Philips Ingenia 3.0T CX. As pulse sequences, applied to the analysis, mDixon (O-MAR), CS-SEMAC (SPIR), and STIR were used. As the coil used to obtain data, the dStream Head Spine Coil was used. When tested directly applying to the transplant patients in the conditions the same as for the Phantom, as for the MAR effect of T1 and T2 images, the SNR value showed the highest effect on the increase in the signal in T1, T2 CS-SEMAC (SPIR), followed by mDixon (O-MAR) and STIR, which was the same result as the Phantom (*p* < 0.05). In addition, in the results of the histogram measurement in both of the subjects, Phantom and transplant patients, the count of T1, the T2 Sagittal image was the highest in T1, T2 STIR, followed by T1, T2 mDixon (O-MAR) and T1, and T2 CS-SEMAC (SPIR). As a result of the qualitative analysis, the quality was the best in T2 CS-SEMAC(SPIR) (c), followed by mDixon (O-MAR) (b) and T2 STIR (a). In conclusion, when the MAR effect on the Pig L-spine Phantom and Transplant patients was compared, it was noted that the CS-SEMAC (SPIR) technique was the most excellent in the following order: STIR < mDixon (O-MAR) < CS-SEMAC (SPIR).

## 1. Introduction

MRI is the process of producing an image, receiving a signal coming from the protons by causing a magnetic resonance phenomenon using radio frequency (RF) in the atomic nucleus of the tissue in the body [1] and plays a leading role in the diagnosis and treatment process of clinical medicine without any radiation exposure and with excellent tissue-resolving power and spatial resolution [2]. MRI is patient-friendly testing that can be utilized for the non-invasive and iterative quantification of the real fat content in bone marrow and muscle in the same volume [3]. In addition, it is possible to diagnose diseases and detect the anatomical location of lesions more easily by realizing the 3D image of the structure of the human body, which is hard to see directly, and especially, it is employed as one of the ideal methods of inspection in musculoskeletal tests.. However, in MRI, various artifacts that disturb the accurate diagnosis are produced. The most common one of them is the magnetic susceptibility artifact, and in particular, metal artifacts by metal [4]. Metal artifacts jeopardize the image diagnosis and post-operative evaluation of patients. Moreover, with the development of modern medicine, treatment using various ferromagnetic implants is increasing [5]. In particular, in the orthopedic surgery field, as the insertion of metal objects into the human body increases, it is necessary to diagnose the surroundings of metal implants frequently, so that removing metal artifacts from MRI images stood out as an important issue [6]. Thus, to reduce the metal artifacts that reduce diagnostic value and the homogeneity of the magnetic field and distort the images, various Metal Artifact Reduction (MAR) techniques were developed. Of the MAR techniques, the Dixon technique is one of the Fat Suppression (FS) techniques, which can separately reconstruct pure water and fat images by leveling off the data of the sum and difference of the water signal and fat signal, using the phase shift between water and fat [7,8]. Philips’s Dixon technique, mDixon, is an abbreviation of modified Dixon, which can freely set TE through calculation without setting TE for the in-phase and out-of-phase.

An in-phase signal is the value of the arithmetic operation of the sum of Fat Signal (F) and Water Signal (W) and Systemic Error Phase (∅), and out-of-phase signal is the value of the arithmetic operation of Systemic Error Phase (∅) and Magnetic Field Error Phase (∅_0) with the difference between Fat Signal (F) and Water Signal (W). Here, mDixon can draw the fat signal value and water signal value, respectively, using the two-phase signals (Equation (1)) [9,10,11,12]:

$$S_{0\;}=\left(W+F\right)\cdot \;e^{i\emptyset_{0}},\;S_{1}=\left(W\;-\;F\right)\cdot \;e^{i\emptyset_{0}}\cdot e^{i\emptyset },\;W=0.5\cdot \left|S_{0}+S_{1}\right|,$$

(1) $$F=0.5\cdot \left|S_{0}-S_{1}\right|,$$

($S_{0}$: In-phase Signal, $S_{1}$: Out-of-phase Signal, W: Water Signal, F: Fat Signal,

$\emptyset_{0}$: Magnetic Field Error Phase, ∅: Systemic Error Phase, e: Napier’s Number).

Thus, unlike other FS techniques that directly apply the fat signal frequency, since it obtains fat suppression images through a mathematical calculation, it is less affected by heterogeneity by the metal artifact. According to a recent study, Short Tau Inversion Recovery (STIR), the FS technique using Inversion Recovery (IR), can obtain high signal intensity even in MRI images in which there are metal implants and reduce image distortion by metal artifacts, so its usefulness is proven [13]. The O-MAR (Orthopedic Metal Artifact Reduction) technique mixed with the mDixon technique is Philips’s MAR technique, which is a method to minimize the distortion by metal materials by additionally applying View Angle Tilting (VAT) to the Turbo Spin Echo (TSE) technique [14]. Second, the Slice Encoding for Metal Correction (SEMAC) technique is a method of correcting the artifact on the plane image by obtaining three-dimensional complex data, applying additional encoding to the phase-coding direction perpendicular to the zaxis based on VAT. In addition, it is compatible with Compressed SENSE (CS), is less sensitive to noise compared to the existing SENSE technique, and has benefits, e.g., it can decrease test time, increase scan coverage, and increase resolution [15,16,17,18,19].

The multi-spectrum MRI technique for MAR, such as SEMAC and MAVRIC, is an advanced technology that can visualize the bone and soft tissue around metal materials by minimizing the artifacts in the In-Plane Image [20,21]. The images reconstructed with the SEMAC technique have relatively lower signal to noise ratio (SNR), and those with MAVRIC may cause aliasing in in-plain image since they lack selectivity for slices in the z-axis direction. To compensate for these shortcomings, the MAVRIC-SL technique, which combines SEMAC and MAVRIC, is also used in the clinic. However, the SEMAC technique applied to this study is the technique that came out for the latest equipment, which supplements the shortcomings. Third, the STIR (Short TI (tau) inversion recovery) technique uses Inversion Time (TI) from the 180° RF pulse to the 90° RF pulse, and there is a little difference in fat TI value in the human body part; however, it is clinically used in the range of approximately 160–180 ms in 3.0T MRI model. At this time, TI can be calculated with the following equation: TI null = In2 × T1 or 0.69 × T1. Currently, as a technique, less clinically affected by metal artifacts, the STIR Fat Suppression (FS) technique mainly includes musculoskeletal testing, which is used as an FS technique for the case in which there are metals in the human body [22]. In the same way as this, studies have been conducted on the metal artifact suppression ability of various techniques developed for MAR [23]; however, since there is insufficient research information on evaluation based on the actual animal Phantom and transplant patients in comparative studies of the effect of MAR between the techniques, it would be necessary to compare them. Thus, this study would evaluate the clinical usefulness of the images obtained after applying mDixon (O-MAR), CS-SEMAC (SPIR), and STIR techniques to Pig L-spine Phantom and transplant patients according to the difference in the reduction in metal artifacts and provide the optimal MAR image technique.

## 2. Subjects and Methods

### 2.1. Subjects

This study was conducted with Pig L-spine Phantom and 30 transplant patients who had an implant on L-Spine (22 men, 8 women, mean age: 64.2 ± 12.98) (Table 1). All of the data analyzed were evaluated, using Philips Ingenia 3.0T CX (Philips Medical System, Amsterdam, The Netherlands). As pulse sequences, applied to the analysis, mDixon (O-MAR), CS-SEMAC (SPIR), and STIR were used. As the coil used to obtain the data, dStream Head Spine Coil was used. All of the patient data were approved by the Subcommittee of the Institutional Bioethics Committees of Cheongju University, concerning the patient data (IRB NO. 1041107-202204-HR-002-1) and all of the patients signed a consent form. The test parameters applied to Pig L-spine Phantom and transplant patients are shown in Table 2.

### 2.2. Process of Producing Phantom

For the evaluation of metal artifact reduction, Pig L-spine Phantom was produced. To reduce errors in data evaluation, the spine region of a pig, which is the most similar to the human L-spine, was chosen. Regions L1 through L4 were designated on the pig’s spine to produce the Phantom by fixing four titanium screws for orthopedic surgery (Ti, Atomic Weight 47.90, Atomic Number 22) to L1, L2, L3, and L4 (Length 3 cm, Diameter 3.5 mm for L1;; Length 3 cm, Diameter 3.5 mm for L2; Length 4.5 cm, Diameter 3.5 mm for L3; and Length 5 cm, Diameter 5 mm for L4).

**Figure 1:**
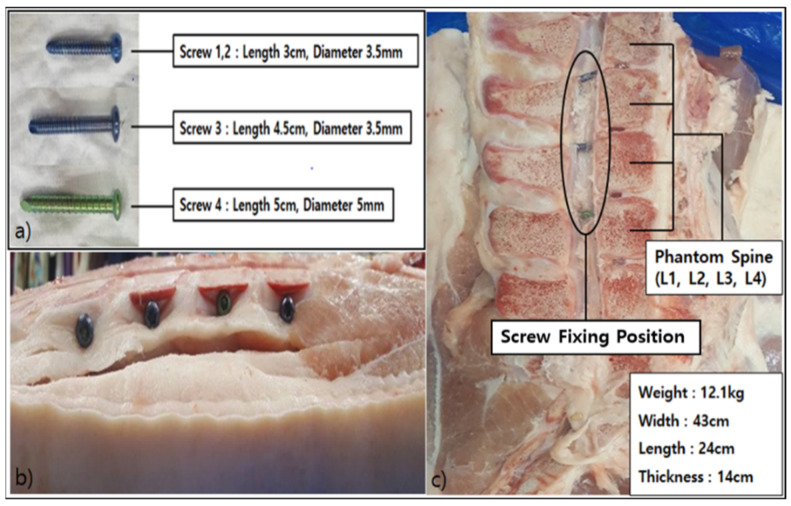
Production of Pig L-spine Phantom. This shows the overall shape of fixation of the screw position in the side of L1–L4 and the sagittal plane. Screw shape and dimensions (**a**); Image of the side of the region where the Screw is fixed (**b**); Dimensions of the Phantom and the sagittal section in which the Screw is fixed by designating L1, L2, L3, and L4 (**c**).

As for the dimensions of the Phantom, it weighed 12.1 kg, was sized 43 cm wide, 24 cm high, 44 cm long, and 14 cm thick. (a) Shape and dimensions of each screw used to produce the Phantom; (b) Left side image of the Phantom to which the screws are fixed; and (c) Dimensions of the Phantom and the image of the spine to which the numbers, L1, L2, L3, and L4, were designated and in which the screws were fixed to the points (Figure 1).

### 2.3. Analysis Method

#### 2.3.1. Quantitative Analysis Method

All of the images obtained for this study were transmitted to Picture Achieving Communication System (PACS), INFINITT Healthcare in a Digital Imaging and Communication in Medicine (DICOM) file format to analyze and evaluate the data, and for all quantitative analyses, ImageJ (Version 1.52p, National Institutes of Health, Bethesda, MD, USA) was used, and the SNR and Histogram Count were measured by the quantitative analyses.

**Figure 2:**
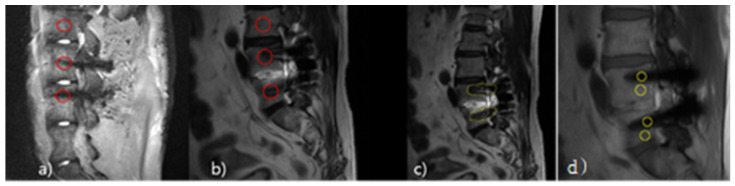
Location of the region of interest (ROI) in Pig L-spine Phantom and transplant patients. Pig L-spine Phantom ROI (**a**); Transplant patient ROI (**b**); Transplant patient Histogram ROI (**c**); Transplant patient CNR ROI (**d**), Circle: Signal Intensity.

As for the SNR measurement, after setting the Region of Interest (ROI), respectively, on the spine body where the metal artifacts were produced and the spine body without distortion at L2, L3, and L4 randomly designated on the Phantom and L3, L4, and L5 of the transplant patients (Figure 2), SNR was measured by calculating the Standard Deviation (SD) of Background Noise, selecting the Signal Intensity (SI) of the ROI and the ROI of the four edges of the image (Equation (2)). CNR was calculated by dividing the difference between the signal in the spinal body area and the signal intensity of the artifact center to it by the mean of the SD of the background signal intensity of the image (Equation (3)).

(2) $$SNR=\frac{{\mathrm{Signal}}_{\mathrm{spine}\;\mathrm{body}}}{\sigma_{\mathrm{Background}\;\mathrm{noise}}},$$

(3) $$CNR=\frac{{\mathrm{Signal}}_{\mathrm{spine}\;\mathrm{body}}-{\mathrm{Signal}}_{\mathrm{arifact}\;\mathrm{center}}}{\rho_{\mathrm{background}\;\mathrm{noise}}}$$

As for the histogram measurement method, the Pig L-spine Phantom and transplant patients’ data were used. All of the data were transmitted to ImageJ in the DICOM file format sent to PACS to conduct a histogram analysis. Centering around the Shadow (dark) section and the Highlight (bright) section of the histogram, the difference in metal reduction was measured. As for the measurement, in L3 and L4 in the 6th Sagittal image of the Pig L-spine Phantom and L4 and L5 of the image of the transplant patient, the count representing the length and height of distortion was measured by setting ROI in the distortion range produced by the metal artifacts based on the dark section and the bright section from the observer’s view.

#### 2.3.2. Qualitative Analysis Method

As for the qualitative analysis method, the images obtained from the three MR techniques were independently evaluated, respectively, by one musculoskeletal radiologist with more than 10 years of experience and one international professional radiological technologist with more than 20 years of MRI experience at a tertiary medical institution.

They evaluated 12 consecutive sagittal plane images for each sequence from a metallicity perspective. The evaluations gave scores in five grades for Overall Image Quality, Susceptibility Artifact, and Pedicle Visualization. The classification levels were 1 for unacceptable, 2 for poor, 3 for fair, 4 for good, and 5 for excellent, and the scores obtained for each image were leveled for comparison. Overall Image Quality was evaluated as follows: 1 point for little metal reduction and visualization of less than 1/3 of the spine body in the region of the screw insertion; 3 for moderate metal reduction and visualization of 1/3–2/3 of the spine body in the region of the screw insertion; and 5 for clear metal reduction and visualization of more than 2/3 of the spine body. For the Susceptibility Artifact, 1 point for clear distortion of CSF in the region of the screw insertion and the surrounding tissue; 3 for less distortion of CSF in the region of the screw insertion and the surrounding tissue; and 5 for vague distortion of CSF in the region of the screw insertion and the surrounding tissue. For Pedicle Visualization, 1 point for obscure Pedicle visualization; 3 for less than 1/3 Pedicle visualization; and 5 for more than 2/3 Pedicle visualization, and in all of the items, 2 and 4 points were given by the evaluator’s subjective evaluation.

### 2.4. Statistical Analysis

A one-way RM ANOVA test was used for the quantitative analysis of three MRI pulse sequence types, and a Bonferroni correction method was used for the post-hoc analysis. For qualitative analysis, the image quality was evaluated using a Friedman test, and the Wilcoxon signed-rank (c > a > b) method was also used for the post-hoc analysis of the Friedman test. A Cohen’s Kappa coefficient greater than 0.6 was judged to be the consistent measurement result. For the quantitative analysis, ImageJ Ver.1.52(National Institutes of Health, Bethesda, MD, USA) was employed, and statistical significance was put to a *p* value lower than 0.05. As for the software used for data analysis, statistical analysis was conducted using the SPSS Software (SPSS 24.0 for Windows; SPSS Inc., Chicago, IL, USA).

## 3. Results

### 3.1. SNR Result of Pig L-Spine Phantom

The pulse sequences applied to the produced Phantom were T1, T2 mDixon (O-MAR), T1, T2 CS-SEMAC (SPIR), and T1, T2 STIR, all of which showed statistically significant results, and in the results, the FS technique was included in all of the techniques (*p* < 0.05). In the SNR measurement, the mean and standard deviation were 21.83 ± 0.42 in the T1, T2 CS-SEMAC (SPIR) technique, which was significantly higher than the other two techniques (14.73 ± 0.18 and 8.23 ± 0.10) (*p* < 0.05) (Table 3). Compared to L2 less affected by metal artifacts by Phantom Screw, the SNR values of L3 and L4 more affected were lower, and the range of distortion was larger. The MAR result was the highest in T1, T2 CS-SEMAC (SPIR), followed by T1, T2 mDixon (O-MAR), and the T1, T2 STIR, and SNR values were also measured to be the lowest in STIR.

### 3.2. SNR Result of Transplant Patients

When the images were evaluated by setting parameters in transplant patients in the conditions the same as for the Phantom, there were significant results as the SNR values increased (*p* < 0.05). In the SNR measurement, it was the highest in the T1, T2 CS-SEMAC (SPIR) technique with the mean and standard deviation of 20.67 ± 0.24 compared to the other two techniques (12.13 ± 0.29 and 8.06 ± 0.15), and there were statistically significant differences (*p* < 0.05) (Table 4). When tested directly, applying to the transplant patients in the conditions the same as for the Phantom, as for the MAR effect of the T1 and T2 images, the SNR value showed the highest effect on the increase in the signal in T1, T2 CS-SEMAC (SPIR), followed by mDixon (O-MAR) and STIR, which was the same result as the Phantom. As the T1, T2 CS-SEMAC (SPIR) technique had the strongest MAR function, the SNR value was the highest. For effective evaluation, this study selected L2, L3, and L4 as the positions of the images for Phantom study and L3, L4, and L5 for the implant study in evaluating SNR with Phantom and transplant patients. Since the Phantom has the spine body up to L6, this resulted from an analysis of the broad regions by crossing L3 and L4 at the L-Spine isocenter in the MAR evaluation, and it was noted that there was no big difference in the signal intensity with the recession of L-Spine from the FOV Isocenter. The result of CNR centered around L4 and L5 from transplant patients was higher in T1, T2 CS-SEMAC (SPIR) mDixon (O-MAR) than in STIR as in the results of the Phantom test. This result is a quantitative data value excluding all of the factors that may be produced from the patients, including artifacts (Table 5).

### 3.3. Histogram Measurement Results

**Figure 3:**
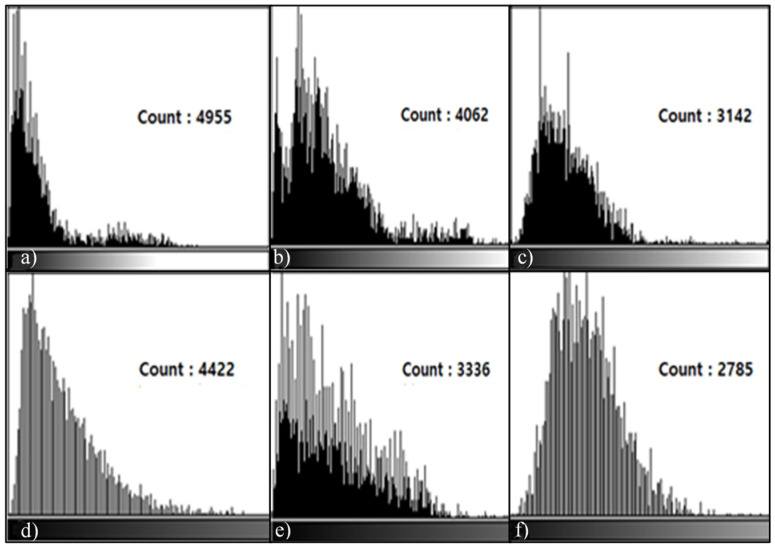
L3, L4 Histogram values measured in the T1 Sagittal Image of Pig L-spine Phantom. When the distribution value was measured in the T1 Sagittal Image at the 6th section, the value was higher in T1 STIR than in T1 CS-SEMAC (SPIR) value, and the signal loss was also higher. T1 STIR Histogram (**a**,**d**); T1 mDixon (O-MAR) Histogram (**b**,**e**); and T1 CS-SEMAC (SPIR) Histogram (**c**,**f**).

**Figure 4:**
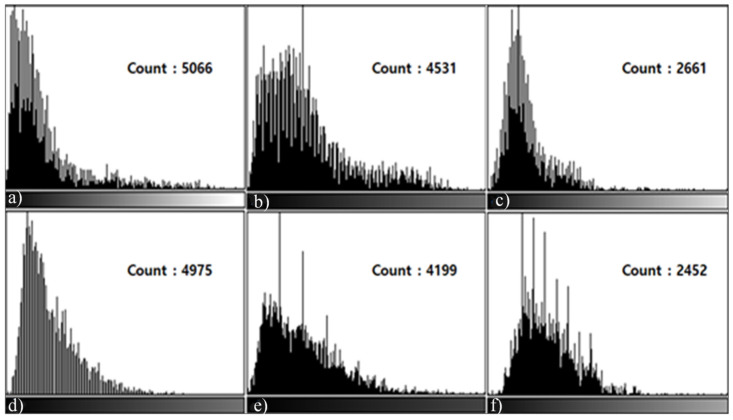
L3, L4 Histogram values measured in the T2 Sagittal Image of Pig L-spine Phantom. When the distribution value was measured in the T2 Sagittal Image, the result was the same in T2 CS-SEMAC (SPIR) as in T1 CS-SEMAC (SPIR). T2 STIR Histogram (**a**,**d**); T2 mDixon (O-MAR) Histogram (**b**,**e**); and T2 CS-SEMAC (SPIR) Histogram (**c**,**f**).

**Figure 5:**
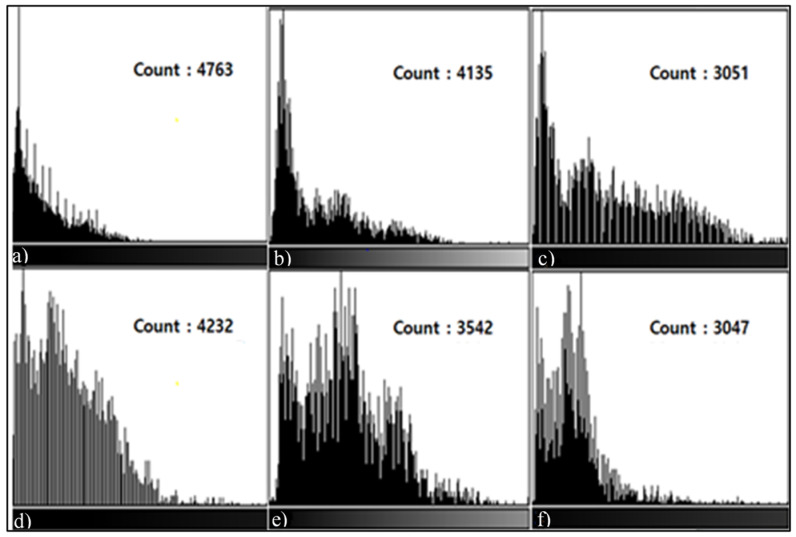
L4, L5 Histogram values measured in the T2 Sagittal Images of transplant patients. In the L4, L5 T2 Sagittal Images, as the same result as that for the Pig L-spine Phantom, the T2 CS-SEMAC (SPIR) technique shows higher values. T2 STIR Histogram (**a**); mDixon (O-MAR) Histogram (**b**); T2 CS-SEMAC (SPIR) Histogram (**c**); and T2 STIR Histogram measured in L5 (**d**); T2 mDixon (O-MAR) Histogram (**e**); and T2 CS-SEMAC (SPIR) Histogram (**f**).

In L3, L4, and L5 Sagittal Histogram Count measured in the T1, T2 Sagittal images, there were differences between the testing techniques. In the results of the histogram measurement of the T1, T2 Sagittal images of the Phantom, the count (L3: 4955, 5066 L4: 4422, 4975) was the highest in T1, T2 STIR, followed by T1, T2 mDixon (O-MAR), and T1, T2 CS-SEMAC (SPIR). Viewed from the histogram graph, in the bright section on the left, there was almost no graph while in the dark section on the right, there was a high graph formed. Overall, the dark section on the right means signal loss due to MAR, and the MAR level of each could be checked by the difference in the count of the graph (Figure 3 and Figure 4). In addition, for the transplant patients, centering around the T2 Sagittal image, in the results of the histogram measurement, the count was extremely high in T2 STIR (L4: 4763, L5: 4232), followed by T2 mDixon (O-MAR), and T2 CS-SEMAC (SPIR). In addition, in the image in Figure 5, the histogram formed a high graph on the right side, and the MAR level could be checked by count.

In addition, in the results of the histogram measurement in both of the subjects, Phantom and transplant patients, the count of the T1, T2 Sagittal image was the highest in T1, T2 STIR, followed by T1, T2 mDixon (O-MAR), and T1, T2 CS-SEMAC (SPIR). According to the results of the histogram measurement, the range of distortion was the widest in T1, T2 STIR while it was the narrowest in T1, T2 SEMAC (SPIR).

### 3.4. Qualitative Analysis Results

Significant results were obtained with qualitative evaluations of Phantom and transplant patients in this study (*p* < 0.05). Table 6 and Table 7 show the results of analysis of T1, T2 Sagittal images to which the MAR and the FS technique were applied to Phantom and transplant patients. For overall image qualities, susceptibility artifact, and pedicle visualization, the T1, T2 CS-SEMAC (SPIR) technique had the highest mean and standard deviation of 4.15 ± 0.05 (*p* = 0.00017). In musculoskeletal disorders, generally, implant transplant patients often use the T2 FS Image (Fat Suppression Image) as a routine protocol in the clinic, thanks to the fact that it detects disorders well. Considering this, in a comparative analysis of T2 Sagittal images, the T2 SEMAC (SPIR) technique showed the highest value of 4.10 ± 0.09. It was a clinically useful result concerning information about T2 Sagittal Fat Suppression in musculoskeletal testing (*p* = 0.0003).

In the image evaluations of each technique and function, the mean was higher than three points (Fair), which was relatively fair. The T2 CS-SEMAC (SPIR) image had higher than four points (good delineation) in all of the items, which most clearly described the contact point between the skeleton and the implant and had the least image distortion and clearest visualization. On the other hand, in the T2 STIR Image, the range of distortion due to the susceptibility artifact was the broadest, and pedicle visualization and the overall image lesion delineation were relatively lacking. In particular, the patients’ T2 STIR sequence image received the lowest evaluations in susceptibility artifact and pedicle visualization, with points lower than 3.

**Figure 6:**
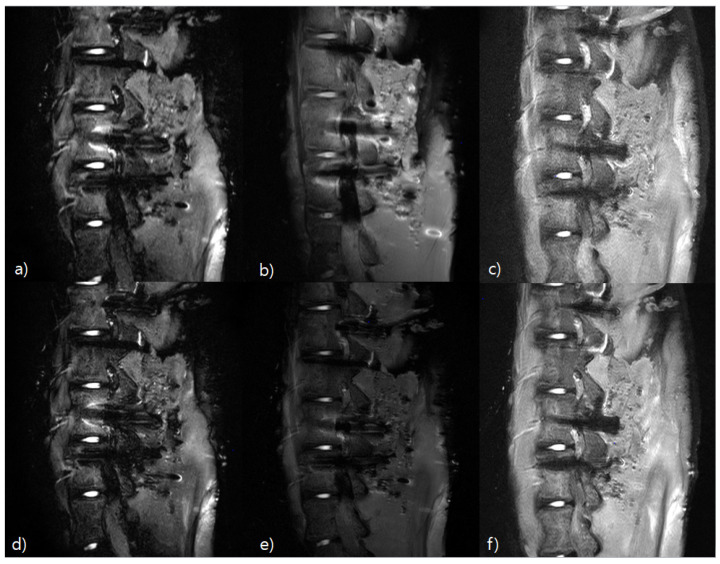
Evaluation of T1, T2 image qualities using Pig L-Spine Phantom. Visually, compared to the other two techniques, T1, T2 SEMAC (SPIR) has a brighter signal intensity and higher MAR effect. T1 STIR Image (**a**); T1 mDixon Image (**b**); T1 CS-SEMAC Image (**c**); T2 STIR Image (**d**); T2 mDixon Image (**e**); and T2 CS-SEMAC Image (**f**).

Figure 6 compares the images obtained with the T1, T2 STIR (a,d), T1, T2 mDixon (O-MAR, b,e), and T1, T2 CS-SEMAC (SPIR, c,f) techniques, using the Phantom. For image qualities, overall, there were fewer metal artifacts and clear distinction of the spine body more than two/thirds in (c) and (f), followed by (b) and (e), (a) and (d). For susceptibility artifact, with the (a) and (d) techniques (STIR), the levels of distortion of CSF and surrounding tissue were severe, so anatomical distinction was more difficult than the other techniques. For pedicle visualization, it was the least in the STIR (a,d) technique due to the screw, and with the CS-SEMAC (SPIR) technique (c,f), the spine body was observed well, more than two/thirds. Of the three techniques, in the SPIR technique, visually, the range and length of the image distortion were large. This result is the same as that showing a high count in the histogram measurement.

Table 8 separates the difference in signal intensity between the spinal body and artifact parts with a transplant patient. In the artifact part, the CS_SEMAC (SPIR) technique had the lowest SRR (%) value (L4: 56.84, L5: 54.59) while STIR had the highest value (L4: 72.45, L5: 66.33).

**Figure 7:**
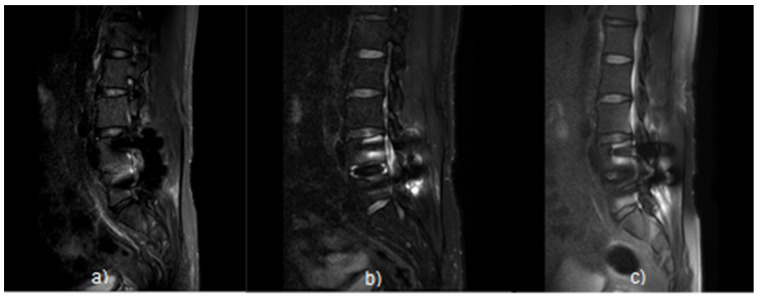
L4, L5 L-Spine Sagittal image of patientswho had transplant surgery. In the T2 Sagittal Images, the T2 CS-SEMAC (SPIR) Fat Suppression image has an excellently less signal loss compared to T2 STIR Fat Suppression. T2 STIR (**a**); T2 mDixon (O-MAR) (**b**); and T2 CS-SEMAC (SPIR) (**c**).

The T2 STIR (a), T2 mDixon (O-MAR) (b), and T2 CS-SEMAC (SPIR) (c) images were comparatively evaluated with the L-Spine Sagittal images of patients who had implant surgery in L4 and L5 (Figure 7). The image of the (c) technique in Figure 7 overall had excellent results with a higher MAR effect than the (a) and (b) techniques and clearer distinction of CSF and the spine body. In particular, in the (c) image, the lesion delineation of the contact point between the implant and the pedicle is the clearest, has less range of distortion, and excellent overall visualization. As a result of the qualitative analysis, the quality was the best in the T2 CS-SEMAC (SPIR) (c), followed by the mDixon (O-MAR) (b), and the T2 STIR (a).

### 3.5. Results of Post-Hoc Analysis and Cohen’s Kappa Analysis

In the observer accuracy evaluation of ROI in the images from which the data were obtained, the Kappa coefficient was 0.8–0.9, showing high consistency. As a result of post-hoc analysis in the Sagittal images of the Phantom and transplant patients, there were significant differences in both mDixon (O-MAR) and STIR based on L1, L2 CS-SEMAC (SPIR), and the other techniques had the same results (*p* < 0.005). As a result of the analysis, it was found that the L1, L2 CS-SEMAC (SPIR) technique had the most excellent fat suppression and MAR effect.

## 4. Discussion

Magnetic susceptibility that occurs in MRI testing refers to that of the subject in the magnetic field. Since the magnetic susceptibility of metal is much more sensitive than human tissues, the Larmor precession frequency changes are greater around metal, and accordingly, the signal decreases and loss takes place [24]. Changes in the magnetic field by metal produce artifacts in both the Through-plane and In-plane and accordingly produce various image distortions, such as Signal Pile-up, Curved Slice and abnormally thick or thin Slice, and Split Slice, in addition to signal loss. This stood out in particular as an important issue in the orthopedic surgery field that uses ferromagnetic material implants in various regions such as the cervical spine, lumbar spine, knee, and hip, etc., and accordingly, it would be necessary to develop various MAR functions, such as SEMAC and O-MAR, etc. [25]. This experiment evaluated the MAR function, applying techniques to Phantom and transplant patients to solve the problems with these ferromagnetic material-related metallic materials.

The reason why the Pig Phantom was used in this study is that the enlargements in pigs and humans are largest and most similar in size (length and cross-sectional area); followed by monkeys [26]. It has an advantage that it obtains constant data on all phenomena that may occur, including artifacts, which may be produced from the patient.. Specifically, despite this advantage, it is impossible to find the exact center frequency in fat suppression in the part in which there is metal since there is a severe change in the resonance frequency.. Thus, this Phantom test provided the optimum information for the quantitative evaluation of three fat suppression technique types in which there was metal.

When the images were obtained, the CS-SEMAC (SPIR) technique had a higher MAR effect than the other techniques, but the mDixon (O-MAR) and STIR techniques showed weak differences in the images, so it would be necessary to adjust the parameters.

In the parameters, the bandwidth was set as high as possible, and the protocol was set with a TE smaller than the value applied in the clinic (mDixon: 18 ms, 120, STIR: 60 ms, 110 ms). Since small slice thickness (thin slices) also has a high MAR effect, it was changed from 4 mm, Gap 0.4 mm, to 3 mm, Gap 0.3 mm. In addition, as a method for obtaining parallel imaging data, the recently developed Compressed SENSE technique was applied, not the existing method, SENSE (Sensitivity Encoding) technique [27]. With the adjustment of the Compressed SENSE factor, it was possible to reduce the inspection time by 25% and decrease the patients’ motion artifacts, and there was an effect on the increase in the spatial resolution around the L-spine body and in the CSF and spinal cord to the maximum. The issue of Specific Absorption Rate (SAR) generated in 3.0T high field magnetic could be reduced to some extent, and especially because it reduced the quantity of RF that was generated in the Phased-Array Coil, it served to a small degree as MAR [28]. 

In the data analysis, with the SPIR, STIR, and MAR techniques related to fat suppression, a strong gradient is applied in obtaining images, and eddy current artifacts may be produced. Eddy current artifacts become the cause for image distortion by the induced currents. As a result, with a fast T2* decrease (decay), a signal decrease occurs, which appears severer as the FOV deviates from the center of the magnet. In addition, in the author’s study, the Phantom study selected the range L2–L4, and the Implant Study, L3–L5. One of the reasons for choosing the broad-crossing ranges was to evaluate the impact of eddy current artifacts. Since the L-Spine image acquisition uses a phased array coil, much heat is generated, which is related to the image distortion along with the risk of burning of the patient in the MRI bore. In addition, since there are previous studies that reported that it caused this risk for patients in the 3.0T high magnetic field, MRI users should use caution to position the location [29]. In this study, too, Active Gradient Shielding Coil performance and Gradient Shield were checked to reduce the eddy current artifacts before testing.

**Figure 8:**
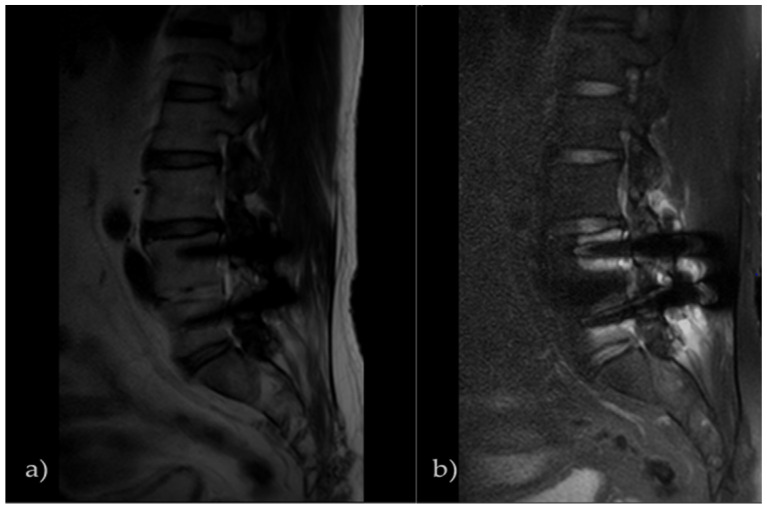
T2 CS-SEMAC image of transplant patients. In musculoskeletal testing, if there is a metal material, the image to which the fat suppression technique was applied shows a great signal loss. This is an image that compared Image (**a**) to which the FS technique was not applied and Image (**b**) to which it was applied. MAR was less in Image (**a**) than in Image (**b**).

The existing report reported on the SEMAC + VAT technique [30]. However, this study analyzed Phantom Imaging only, not transplant patients, did not apply the FS technique to which importance is attached in the musculoskeletal system, and did not apply the recently developed Compressed SENSE (CS) technique. In addition, a study that also compared the conventional FS technique, using the mDixon and SPIR techniques noted that the two techniques had the same or a little higher result. It was reported that this result would clinically be considered in terms of the qualitative aspect of images [31]. However, since this study completed the FS evaluation, using the recently released 3.0T equipment and CS-SEMAC (SPIR) technique suitable for the latest MRI models, it provided lots of information about the MAR effect. For transplant patients, combining MAR and the FS technique is very important for the accurate diagnosis of bone marrow and soft tissue [32]. If there is metal in the human body part, to obtain images without metal distortion, the T2 CS-SEMAC technique not applying the FS Technique would be much more helpful in musculoskeletal interpretation. As shown in Figure 8, it is an image by which the level of the artifacts decrease in the metal materials can be checked with the T2 CS-SEMAC (a) and T2 CS-SEMAC (SPIR) (b) technique that used the FS technique. Comparing the L4, L5 spine body (a,b), it is noted that in Image (a), the metallic materials by the screw invaded less in the spine body. From this result, it is judged that obtaining images not applying the FS technique additionally after testing with the MAR technique would be useful for interpretation. A contrast medium is a material that affects MAR function. The contrast medium is a paramagnetic substance, which causes the signal increase by shortening the relaxation time of T1 by the local magnetic field. At this time, if there is metal around a blood vessel, the difference in magnetic susceptibility becomes severer, so that severe image distortion takes place. Thus, if the T1 weight image (WI) is obtained after injecting the contrast medium, testing without applying the FS technique would be an effective test for interpretation. According to the author’s experience, currently, tests with or without FS in musculoskeletal testing are interpreted in radiology. To sum up the MAR techniques, the STIR technique and the Dixon technique have long been applied to MRI interpretation, and later, the mDixon technique has much been used as a MAR technique, but there was a demand for a better MAR effect. The CS-SEMAC technique mostly applied presently could supplement that, and the 3.0 T high magnetic field equipment provided reasonable information as a high MAR technique.

As a limitation, in applying the FS to the CS-SECMAC (SPIR) technique, the images were obtained by combining it with SPAIR and STIR concretely, with various FS techniques provided by Philips. However, it is judged that it could be supplemented, since the technique used in the research was set to the combination recommended by Philips.

## 5. Conclusions

In conclusion, when the MAR effect on Pig L-spine Phantom and transplant patients was compared, it was noted that the CS-SEMAC (SPIR) technique was the best in the following order: STIR < mDixon (O-MAR) < CS-SEMAC (SPIR). In particular, using Compressed Sense as a Parallel Imaging Technique with each technique increased the MAR effect, the SNR, the CNR and the resolution, and that played a role in decreasing the scan time by more than 25% to be able to reduce the patient’s motion artifact.

## Figures and Tables

**Table 1:** Main features of the 30 study transplant patients.

Features	Mean ± SD/(%)
Age (years)	64.23 ± 12.98
Males/Females	22 (73.33%)/8 (26.66)
Height (m)	1.6 ± 0.2
Weight (kg)	68.25 ± 15.32
Osteoporosis	13 (43.33%)
DDD	15 (50%)
IS	2 (6.66%)
LT	30 (100%)

SD: Standard deviation; DDD: Degenerative disc disease; IS: Isthmic spondylolisthesis; LT: L-spine Transplant.

**Table 2:** T1, T2 Scan Parameters six kinds of MAR effect were applied to Phantom and patients.

Parameters	T1 mDixon	T1 STIR	T1 SEMAC	T2 mDixon	T2 STIR	T2 SEMAC ^(d)^
TR ^(^^a)^ (ms)	558	3110	589	1980	3215	2100
TE ^(^^b)^ (ms)	18	60	50	120	110	110
ST (mm)	3	3	3	3	3	3
Gap (mm)	0.3	0.3	0.3	0.3	0.3	0.3
Matrix	416 × 250	416 × 233	416 × 250	416 × 239	416 × 250	416 × 250
NEX	2	2	2	2	2	2
FOV (mm)	250	250	250	250	250	250
ST ^(c)^	02 : 36	06 : 19	10 : 55	04 : 06	07 : 24	14 : 23

^(a)^ TR: Repetition Time; ^(b)^ TE: Echo Time; ^(c)^ ST: Scan Time; ^(d)^ SEMAC: CS SEMAC.

**Table 3:** SNR result of T1, T2 MAR effect in testing Pig L-spine Phantom.

Units		mDixon (O-MAR) ^a^	STIR ^b^	CS-SEMAC (SPIR) ^c^	*p*-Value
L-2	T1 Sagittal	19.32 ± 0.79	11.20 ± 0.48	28.13 ± 1.24	<0.001, (c > a > b)
	T2 Sagittal	18.49 ± 0.81	9.52 ± 0.42	33.15 ± 1.52	<0.001, (c > a > b)
L3	T1 Sagittal	17.52 ± 0.61	9.59 ± 0.34	22.81 ± 0.96	<0.001, (c > a > b)
	T2 Sagittal	14.23 ± 0.62	6.82 ± 0.27	23.25 ± 1.04	<0.001, (c > a > b)
L4	T1 Sagittal	10.31 ± 0.41	7.46 ± 0.32	10.92 ± 0.45	<0.001, (c > a > b)
	T2 Sagittal	8.51 ± 0.38	4.84 ± 0.17	12.77 ± 0.47	<0.001, (c > a > b)

Note: Numbers are mean ± standard deviation. (one-way repeated measure ANOVA test, post-hoc (c > a > b): Bonferroni). In testing Phantom, applying three kinds of technique, the T1, T2 CS-SEMAC (SPIR) technique had the highest value in L2, L3, and L4 (*p* < 0.05).

**Table 4:** SNR result of T1, T2 MAR effect in testing transplant patients.

Units		mDixon (O-MAR) ^a^	STIR ^b^	CS-SEMAC (SPIR) ^c^	*p*-Value
L3	T1 Sagittal	18.75 ± 0.91	7.80 ± 0.27	32.55 ± 1.12	<0.001, (c > a > b)
	T2 Sagittal	12.20 ± 0.24	9.01 ± 0.58	22.27 ± 0.84	<0.001, (c > a > b)
L4	T1 Sagittal	8.93 ± 0.44	7.58 ± 0.21	14.2 ± 0.65	<0.001, (c > a > b)
	T2 Sagittal	10.42 ± 0.90	8.09 ± 0.52	12.17 ± 0.44	<0.001, (c > a > b)
L5	T1 Sagittal	11.40 ± 0.49	7.69 ± 0.28	22.17 ± 0.89	<0.001, (c > a > b)
	T2 Sagittal	11.09 ± 0.27	8.24 ± 0.47	18.13 ± 0.61	<0.001, (c > a > b)

Note: Numbers are mean ± standard deviation. (one-way repeated measure ANOVA test, post-hoc (c > a > b): Bonferroni). In testing transplant patients, CS-SEMAC (SPIR) technique had the highest value (*p* < 0.05) with T1, T2 MAR effect, which was the same as the result of Phantom testing.

**Table 5:** CNR result of T1, T2 MAR effect in testing transplant patients.

Units		mDixon (O-MAR) ^a^	STIR ^b^	CS-SEMAC (SPIR) ^c^	*p*-Value
L4	T1 Sagittal	7.42 ± 0.20	4.61 ± 0.11	13.28 ± 0.74	<0.001, (c > a > b)
	T2 Sagittal	9.59 ± 0.84	8.34 ± 0.12	19.25 ± 0.48	<0.001, (c > a > b)
L5	T1 Sagittal	10.25 ± 0.50	6.33 ± 0.14	15.13 ± 0.43	<0.001, (c > a > b)
	T2 Sagittal	15.01 ± 0.40	7.47 ± 0.15	17.03 ± 0.30	<0.001, (c > a > b)

Note: Numbers are mean ± standard deviation. (one-way repeated measure ANOVA test, Post-hoc (c > a > b): Bonferroni). In testing transplant patients, CS-SEMAC (SPIR) technique had the highest value (*p* < 0.05) with T1, T2 MAR effect, which was the same as the result of Phantom testing.

**Table 6:** Result of qualitative analysis of T1, T2 MAR effect when tested with Pig L-spine Phantom (Median).

		mDixon (O-MAR) ^a^	STIR ^b^	CS_SEMAC (SPIR) ^c^	*p*-Value
Overall Image Qualities	T1 Sagittal	(2.2–5)	(2–4)	(3.5–5)	<0.001, (c > a > b)
	T2 Sagittal	(2.5–5)	(2.5–4)	(3.5–5)	<0.001, (c > a > b)
Susceptibility Artifact	T1 Sagittal	(2.5–5)	(2.5–4)	(3.8–5)	<0.001, (c > a > b)
	T2 Sagittal	(2.5–5)	(2.5–4)	(3.2–5)	<0.001, (c > a > b)
Pedicle Visualization	T1 Sagittal	(2–5)	(2–4)	(3–5)	<0.001, (c > a > b)
	T2 Sagittal	(2–5)	(2.1–4)	(3–5)	<0.001, (c > a > b)

Note: Numbers are mean ± standard deviation, *p*-value: Friedman test, post-hoc: Wilcoxon signed-rank test (c > a > b). When evaluated in 5 grades (1 point–5 points) for the overall image qualities, susceptibility artifact, and pedicle visualization, the T1, T2 CS-SEMAC (SPIR) technique had the highest score. There were significant differences in the comparison of the three groups (*p* < 0.05).

**Table 7:** Result of qualitative analysis of T2 Fat Suppression MAR effect when tested with transplant patients (Median).

	T2 mDixon (O-MAR) ^a^	T2 CS-SEMAC (SPIR) ^b^	T2 STIR ^c^	*p*-Value
Overall Image Qualities	(2.5–5)	(3.5–5)	(2.5–4)	<0.001, (b > a > c)
Susceptibility Artifact	(2.5–4)	(3–5)	(2–4)	<0.001, (b > a > c)
Pedicle Visualization	(3–5)	(3.5–5)	(2.4–5)	<0.001, (b > a > c)

Note: Numbers are mean ± standard deviation, *p*-value: Friedman test, post-hoc: Wilcoxon signed-rank test (b > a > c). As the same as the result of Phantom when tested using T2 mDixon (O-MAR), T2 SEMAC (SPIR), and T2 STIR techniques, the T2 CS-SEMAC (SPIR) technique had the highest score, which was the same result of the test with the Phantom. Due to the T2 Fat Suppression MAR effect usually used in the musculoskeletal system, there were significant differences among the three techniques (*p* < 0.05).

**Table 8:** The difference in signal intensity between the spinal body and artifact parts with a transplant patient.

		Spine Body	Artifact	Diff.	SRR(%)
mDixon (O-MAR)	L4	59.20 ± 7.25	22.45 ± 4.20	36.75 ± 2.15	65.58
	L5	54.21 ± 5.02	20.10 ± 2.08	33.76 ± 2.07	62.27
STIR	L4	45.10 ± 4.60	13.84 ± 2.03	31.26 ± 1.81	72.45
	L5	48.27 ± 3.10	16.25 ± 2.87	32.02 ± 0.16	66.33
CS_SEMAC (SPIR)	L4	67.74 ± 8.57	29.23 ± 3.57	38.51 ± 3.53	56.84
	L5	70.56 ± 7.41	32.04 ± 2.30	38.52 ± 3.61	54.59

Note: Numbers are mean ± standard. Diff: Difference, SRR (%): Signal Reduction Rate, the lower, the higher the MAR effect.

## Data Availability

All the data presented in this study are available upon request from the first author.

## References

[1] Marques J.P., Simonis F.F.J., Webb A.G. (2019). Low-field MRI: An MR physics perspective. J. Magn. Reson. Imaging.

[2] Cunningham P.M., Law M., Schweitzer M.E. (2006). High-Field MRI. Orthop. Clin..

[3] Zhang Y., Zhou Z., Wang C., Cheng X., Wang L., Duanmu Y., Zhang C., Veronese N., Guglielmi G. (2018). Reliability of measuring the fat content of the lumbar vertebral marrow and paraspinal muscles using MRI mDIXON-Quant sequence. Diagn. Interv. Radiol..

[4] Lee J.-H. (2018). Evaluation of O-MAR XD Technique for Reduction of Magnetic Susceptibility Artifact of Knee Implant. J. Radiol. Sci. Technol..

[5] Talbot B.S., Weinberg E.P. (2016). MR Imaging with Metal-suppression Sequences for Evaluation of Total Joint Arthroplasty. Radiographics.

[6] Kim H. (2013). Quantitative evaluation of MRI distortion using orthopedic prosthetic metal. J. Radiol. Sci. Technol..

[7] Cho Y.B., Sung J.G., Young Y., Yang S.W., Seo D.G. (2015). Study to reduce the swap artifact occurring fat suppression Dixon technique used in the MRI scans through self-correction material. J. Korean Soc. MR Technol..

[8] Ma J. (2008). Dixon techniques for water and fat imaging. J. Magn. Reson. Imaging.

[9] Jung D.B., Lee H.K., Heo Y.C. (2021). Comparison of mDixon, T2 TSE, and T2 SPIR Images in Magnetic Resonance Imaging of Lumbar Sagittal Plane. J. Korean Soc. Radiol..

[10] Park M.C., Lee J.H., Kim K.J., Bae S.H. (2014). Evaluation of Usefulness of an m-DIXON Technique during an Abdomen MRI Examination: A Comparison with an e-THRIVE Technique. J. Digit. Converg..

[11] Perkins T.G., Duijndam A., Eggers H., de Weerdt E., Rijckaert Y.H.E. (2015). mDIXON XD—The Next Generation Fat-Free Imaging. PHILIPS 2015.

[12] Back I.H., Pee W.H., Kim J.D., Lee S.K. (2015). Usability Evaluation of mDixon Technique by Comparing with Fat-suppression Techniques Metal Artifacts. J. Korean Soc. MR Technol..

[13] Lee Y.H., Hahn S., Kim E.J., Suh J.S. (2016). Fat-suppressed MR Imaging of the Spine for Metal Artifact Reduction at 3T: Comparison of STIR and Slice Encoding for Metal Artifact Correction Fat-suppressed T2-weighted Images. Magn. Reson. Med. Sci..

[14] de Cesar Netto C., Fonseca L.F., Fritz B., Stern S.E., Raithel E., Nittka M., Schon L.C., Fritz J. (2018). Metal artifact reduction MRI of total ankle arthroplasty implants. Eur. Radiol..

[15] Reichert M., Ai T., Morelli J.N., Nittka M., Attenberger U., Runge V.M. (2015). Metal artefact reduction in MRI at both 1.5 and 3.0 T using slice encoding for metal artefact correction and view angle tilting. Br. J. Radiol..

[16] Geerts Ossevoort L., de Weerdt E., Duijndam A., van Yperen G., Peeters H., Doneva M., Nijenhuis M., Huang A. Compressed SENSE, Speed Done Right. Every Time. https://philipsproductcontent.blob.core.windows.net/assets/20180109/619119731f2a42c4acd4a863008a46c7.pdf.

[17] Lustig M., Donoho D.L., Santos J.M., Pauly J.M. (2008). Compressed Sensing MRI[A look at how CS can improve on current imaging techniques]. IEEE Signal Process. Mag..

[18] Rani M., DHOK S.B., DESHMUKH R.B. (2018). A Systematic Review of Compressive Sensing: Concepts, Implementations and Applications. IEEE Access.

[19] Lu W., Pauly K.B., Gold G.E., Pauly J.M., Hargreaves B.A. (2009). SEMAC: Slice encoding for metal artifact correction in MRI. Magn. Reson. Med..

[20] Filli L., Jud L., Luechinger R., Nanz D., Andreisek G., Runge V.M., Kozerke S., Farshad-Amacker N.A. (2017). Material-Dependent Implant Artifact Reduction Using SEMAC-VAT and MAVRIC: A Prospective MRI Phantom Study. Investig. Radiol..

[21] Gutierrez L.B., Do B.H., Gold G.E., Hargreaves B.A., Koch K.M., Worters P.W., Stevens K.J. (2015). MR Imaging Near Metallic Implants Using MAVRIC SL: Initial Clinical Experience at 3T. Acad. Radiol..

[22] Kishida Y., Koyama H., Seki S., Yoshikawa T., Kyotani K., Okuaki T., Sugimura K., Ohno Y. (2018). Comparison of fat suppression capability for chest MR imaging with Dixon, SPAIR and STIR techniques at 3 Tesla MR system. Magn. Reson. Imaging.

[23] Toms A.P., Smith-Bateman C., Malcolm P.N., Cahir J., Graves M. (2010). Optimization of metal artefact reduction (MAR) sequences for MRI of total hip prostheses. Clin. Radiol..

[24] Huang S.Y., Seethamraju R.T., Patel P., Hahn P.F., Kirsch J.E., Guimaraes A.R. (2015). Body MR Imaging: Artifacts, k-Space, and Solutions. Radiographics.

[25] Olsen R.V., Munk P.L., Lee M.J., Janzen D.L., MacKay A.L., Xiang Q.-S., Masri B. (2000). Metal Artifact Reduction Sequence: Early Clinical Applications. Radiographics.

[26] Toossi A., Bergin B., Marefatallah M., Parhizi B., Tyreman N., Everaert D.G., Rezaei S., Seres P., Gatenby J.C., Perlmutter S.I. (2021). Comparative neuroanatomy of the lumbosacral spinal cord of the rat, cat, pig, monkey, and human. Sci. Rep..

[27] Morita K., Nakaura T., Maruyama N., Iyama Y., Oda S., Utsunomiya D., Namimoto T., Kitajima M., Yoneyama M., Yamashita Y. (2020). Hybrid of Compressed Sensing and Parallel Imaging Applied to Three-dimensional Isotropic T(2)-weighted Turbo Spin-echo MR Imaging of the Lumbar Spine. Magn. Reson. Med. Sci..

[28] Zhang X., Liu J., He B. (2014). Magnetic-Resonance-Based Electrical Properties Tomography: A Review. IEEE Rev. Biomed. Eng..

[29] Tokue H., Tokue A., Tsushima Y. (2019). Unexpected magnetic resonance imaging burn injuries from jogging pants. Radiol. Case Rep..

[30] Qi S., Wu Z.G., Mu Y.F., Gao L.L., Yang J., Zuo P.L., Nittka M., Liu Y., Wang H.Q., Yin H. (2016). SEMAC-VAT MR Imaging Unravels Peri-instrumentation Lesions in Patients With Attendant Symptoms After Spinal Surgery. Medicine.

[31] Pokorney A.L., Chia J.M., Pfeifer C.M., Miller J.H., Hu H.H. (2017). Improved fat-suppression homogeneity with mDIXON turbo spin echo (TSE) in pediatric spine imaging at 3.0 T. Acta Radiol..

[32] Molière S., Dillenseger J.-P., Ehlinger M., Kremer S., Bierry G. (2017). Comparative study of fat-suppression techniques for hip arthroplasty MR imaging. Skelet. Radiol..

